# Processing and Mechanical Properties of Ti_2_AlC MAX Phase Reinforced AE44 Magnesium Composite

**DOI:** 10.3390/ma13040995

**Published:** 2020-02-23

**Authors:** Xufeng Pi, Wenbo Yu, Chaosheng Ma, Xiaojun Wang, ShouMei Xiong, Antoine Guitton

**Affiliations:** 1 Center of Materials Science and Engineering, School of Mechanical and Electronic Control Engineering, Beijing Jiaotong University, Beijing 100044, China; 19121357@bjtu.edu.cn (X.P.); 19125991@bjtu.edu.cn (C.M.); 2 School of Materials Science and Engineering, Harbin Institute of Technology, Harbin 150001, China; xjwang@hit.edu.cn; 3 School of Materials Science and Engineering, Tsinghua University, Beijing 100084, China; smxiong@tsinghua.edu.cn; 4 Université de Lorraine–CNRS–Arts et Métiers ParisTech–LEM3, 7 rue Félix Savart, Metz 57070, France; 5 Labex Damas–Université de Lorraine, Metz 57073, France

**Keywords:** AE44 alloy, Ti_2_AlC MAX phases, anisotropic

## Abstract

AE44 alloys and nanolaminated Ti_2_AlC particle-reinforced AE44 magnesium composites were synthesized by stir casting techniques and textured by hot extrusion methods. It was found that lamellar Al_11_RE_3_ precipitates spheroidized with the introduction of Ti_2_AlC into the AE44 matrix. Both transmission electron microscope and planar disregistries calculations reveal a good match for interfacial lattice transition between Mg (0001) and the basal plane (0001) of Ti_2_AlC. This suggests that Ti_2_AlC is an efficient potent nucleating substrate for Mg, thus fertilizing the formation of strong interfacial bonds. After hot extrusion treatment, Ti_2_AlC particles were reoriented in the textured magnesium matrix, as confirmed by X-ray diffraction. This texture effect on the composite’s mechanical properties was carefully studied by tensile and compressive tests.

## 1. Introduction

To meet the requirements of reducing the weight of transport tools, especially in automobiles, the lightest structures of magnesium metal are sought as attractive candidates [1,2]. Introducing other elements into Mg alloys using alloy design methods can ameliorate stiffness and mechanical properties of the pure metal at high temperatures. For example, by adding 4% RE (rare earth metal) into Mg alloys to form the AE44 alloy (4% Al and 4% RE) [3,4], the new formed Al_x_Re_y_ (Al_11_Re_3_) phase could stabilize Mg_17_Al_12_ phases at high temperatures [5]. However, the stiffness and wear resistance of magnesium alloys are still relatively low, and the application of Mg alloys into automobile cylinders is still restricted due to requirements of high stiffness, wear resistance, and lubrication capacity at elevated temperatures.

To overcome these restrictions, particularly regarding simultaneously enhancing the stiffness and superior wear resistance of Mg alloys, the onlyffective method is to fabricate magnesium composites by introducing ceramic particles into Mg alloys [6,7,8]. Until now, the most widely-used reinforcements in Mg matrices are SiC [9,10], Al_2_O_3_ [5] and graphite particulates [11,12,13]. SiC and Al_2_O_3_ particles can strongly improve the mechanical properties and wear resistance of the matrix [14,15,16], though the damping and self-lubricating capacities of magnesium composites are reduced [12]. High-damping and layered graphite may improve the damping and self-lubricating capacities of magnesium composites [12,13]. Unfortunately, graphite particulates rapidly degrade when the temperature is above 300 ℃, especially in oxidizing environments. In addition, studies show that cracks generally initiate from interfacial areas in these composites, due to weak interfacial bonding [5,9,10,13].

Thus, high-temperature-stable reinforcing particles exhibiting desirable wear resistance, self-lubrication capacity and wettability with magnesium are crucially desired in magnesium composites. Recently, Anasori et al. and Yu et al. found that Ti_2_AlC-Mg composites have a better damping capacity than ones reinforced by traditional ceramic particles such as TiC or SiC [17,18,19,20,21]. M_n+1_AX_n_ phases (n = 1 to 3, M is a transition metal, A is an A-group element and X is nitrogen or carbon) such as Ti_2_AlC have a nanolaminated structure with a hexagonal lattice [22,23,24]. The primitive cell can be described as a stacking of n M_6_X octahedron layers with a layer of A element. Furthermore, measurements of lattice parameters with numerous methods reveal that MAX phases exhibit elevated high crystalline anisotropy. The c/a ratio is generally higher than 4. MAX phases synthesized by powder metallurgy are polycrystalline bulk samples with random grain orientations [25]. It is commonly observed that during synthesis, grains grow in platelet shapes. Due to high crystalline anisotropy, platelet surfaces are parallel to basal planes. Therefore, projections on the surface are observed as rectangles with high aspect ratios. This penny-shape causes deviations from Schmid’s law [26]. Regarding energy, their plastic deformation behavior is only induced by basal dislocations [27,28]. Furthermore, their inherent nanolayered structure endows these ternary compounds with a unique combination of metal-like and ceramic-like properties [17,27,29]. The MAX phase microstructure results in an exceptional self-lubricating behavior. For example, Ti_2_AlC exhibited a low wear rate (1.8 × 10^−6^ mm/(N·m)) during dry sliding against a steel disk [30]. Such strong anisotropic mechanical and tribological properties were expected in MAX phases [9,29,30,31]. For example, Xu et al. reported that textured Ti_3_AlC_2_ shows the lowest mean coefficient of friction in the surface corresponding to the (0001) plane [31]. Furthermore, our previous study on Ti_2_AlC-AZ91D reveals that extrusion methods could regulate the orientation of Ti_2_AlC in AZ91D magnesium composites. Finally, the textured composite has better wear resistance and self-lubricating capacity when the sliding direction is parallel to the extrusion axis [32].

However, at relatively high loads and speeds, a AZ91D matrix-softening phenomenon happened in the Ti_2_AlC-AZ91D composite [32]. As the AE44 alloy has better-reported wear resistance and mechanical properties than the AZ91D alloy at high temperature [3,4], we chose the AE44 alloy over the AZ91D alloy. The purpose of this composite is for use in automobile cylinders requiring high stiffness, wear resistance, and so on. Further studies should be conducted on this composite before its industrial application, such as in cylinder bores. As one part of our project, we focused on the microstructural and mechanical characterization of the Ti_2_AlC-AE44 composite in this study. In addition, we investigated the influence of hot extrusion on this composite with the comparison of the AE44 alloy. 

## 2. Experimental Methods

### 2.1. Fabrication Procedure

In the application of cylinder bores, the reinforcement volume in composites is generally 15% [5,33]. Hence, we fabricated the composite containing 15 vol.% Ti_2_AlC with semisolid stir casting methods. For comparison, AE44 alloy were also produced; the relative technique details may be found in in our previous publications [31,34]. Briefly, as-cast billets with 60 and 300 mm in diameter and height, respectively, were fabricated through semisolid stir casting under the protection of a mixed SF_6_ and CO_2_ gas atmosphere. During this stage, preheated Ti_2_AlC particles were introduced into the magnesium matrix while the melt was in a semisolid condition under stirring. After casting, they were hot-extruded into thin square bars with an extrusion ratio of 14:1. The density of as-cast Ti_2_AlC-AE44 composite was measured by using Archimedes’ principle.

### 2.2. Microstructure Characterization

X-ray diffraction (XRD) using a Bruker D8 diffractometer (Karlsruhe, Germany) with CuK_a_ radiation was used to perform phase identification. Before scanning electron microscope (SEM) (Zeiss Merlin, Germany) observation, each specimen was ground, then successively polished with different diamond suspensions. To observe Mg grain size, a dilute acetic acid solution was used to etch the samples. The solution was composed of 150 mL anhydrous ethyl alcohol, 50 mL distilled water and 1 mL glacial acetic acid. 

TEM samples were fabricated by focused ion beam (FIB) on a Thermo Fisher/FEI Helios dual beam Nanolab 600i equipped with a Ga liquid metal ion source. Then the sample was studied using high-resolution transmission electron microscopy (HRTEM, FEI, Beijing, China) on a Thermo Fisher/FEI Tecnai G2 F20 operating at 200 kV and equipped with a spherical aberration corrector.

### 2.3. Mechanical Properties

A universal servohydraulic mechanical testing machine (Instron 5600, Norwood, MA, USA) was used to perform tensile and compression tests at a strain rate of 0.5 mm/min at room temperature in air. The test specimens were prepared following ASTM E8M-03 flat specimen standards (Standard Test Methods for Tension Testing of Metallic Materials [Metric]). The tensile specimen dimensions were 18 mm in gauge length, 3 mm in width and 2 mm in thickness. The compressive specimen dimension is Φ8 × 12 mm. For each alloy or composite, the tests were repeated six times.

## 3. Results and Discussion

**Figure 1:**
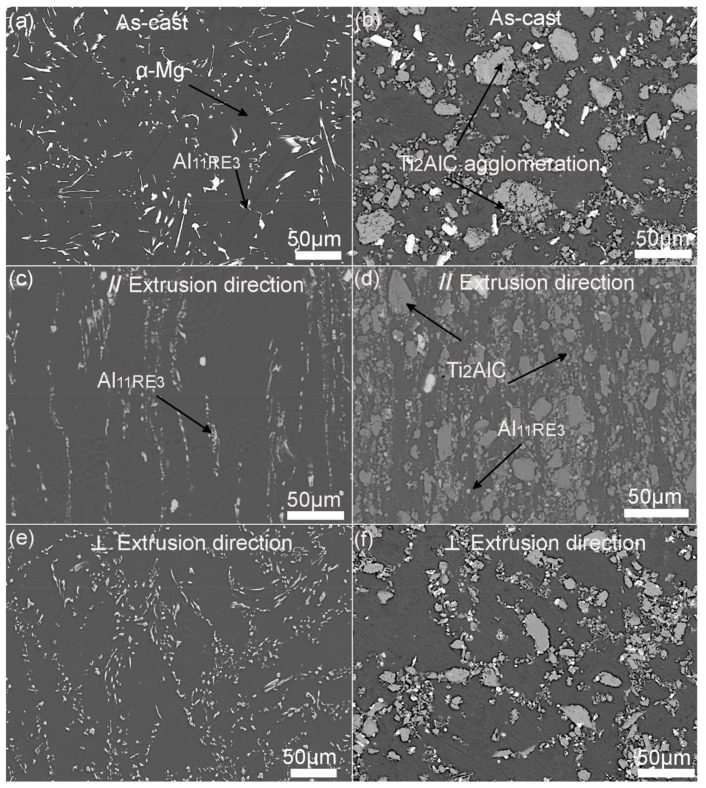
SEM micrographs of AE44 alloys (left column) and 15 vol.% Ti_2_AlC reinforced AE44 composites (right column: (**a**) and (**b**) as-cast, (**c**) and (**d**) // ED(extrusion direction), (**e**) and (**f**) ⊥ ED.

Figure 1a,b represents the microstructure of AE44 alloy (left column) and 15% Ti_2_AlC-reinforced AE44 composite (right column) under backscattered electron (BSE) mode. Figure 1a shows that the lamellar eutectic phases were distributed along the dendritic α-Mg grain boundaries in the as-cast AE44 alloy. Once Ti_2_AlC particles were introduced into AE44 alloy as shown in Figure 1b, the lamellar eutectic phases transformed into round particles. At the same time, Ti_2_AlC particles were relatively uniformly distributed throughout the Mg matrix with some agglomeration. Figure 1c–f presents the different morphologies of extruded AE44 alloy and composites. As reported in metal matrix composites, hot extrusion may result in homogeneous distribution of reinforced particles [35,36]. Regardless of parallel or perpendicular extrusion axis, the accumulated Ti_2_AlC particles along the α-Mg grain boundaries were uniformly redistributed in the Mg matrix. In addition, Al_11_RE_3_ precipitates Al_11_RE_3_ became finer. 

**Figure 2:**
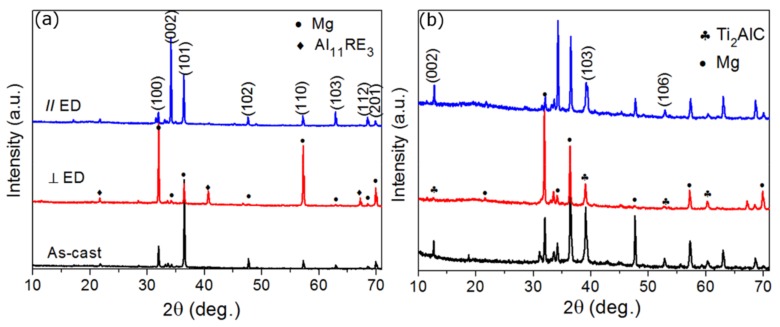
(**a**) and (**b**) XRD patterns of as-cast and hot-extruded AE44 and 15% Ti_2_AlC-AE44 composites.

Figure 2a,b shows XRD results for the as-cast and extruded AE44 and the Ti_2_AlC-AE44 composite. As found in previous study on the extruded Mg [17], the changed intensities in diffraction peaks in Mg indicate that hot extrusion methods can result in a microstructural texture. In Figure 2b, the (002) and (106) diffraction peaks of Ti_2_AlC appeared parallel to ED (extrusion direction), while only very weak traces of these peaks could be retraced perpendicular to the extrusion axis. These results are similar to our previous work on Ti_2_AlC–AZ91D composites [37], which confirmed that the orientation of Ti_2_AlC particles is more easily regulated in magnesium composites duo to its plate-like structure.

**Figure 3:**
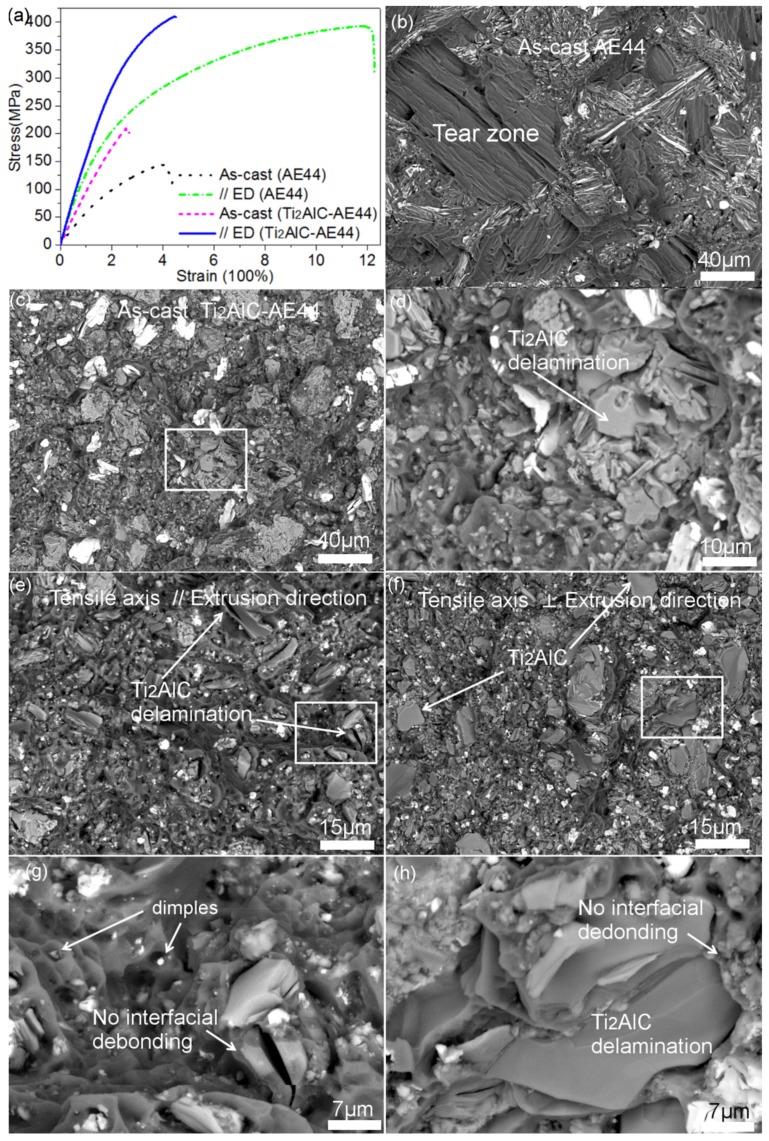
(**a**) Tensile stress-strain curves of the as-cast and extruded specimens (**b**) and (**c**), tensile fracture surfaces of as-cast AE44 and Ti_2_AlC-AE44 composites (**e**) and (**f**), extruded (// ED and ⊥ ED) Ti_2_AlC-AE44 composites (**d**), (**e**), and (**f**), and the enlarged rectangular area marked in (**c**), (**e**), and (**f**).

Figure 3a plots the tensile stress–strain curves of the as-cast and hot extruded specimens (// ED axis). It is evident that the introduction of Ti_2_AlC can efficiently enhance the stiffness of AE44 alloys and hot extrusion can strongly optimize tensile strength. As shown in Table 1, the yield strength of the AE44 alloy was respectively enhanced from 88 to 179 MPa, and from 250 MPa with the introduction of 15 vol.% Ti_2_AlC and hot extrusion treatments. Notably, the yield strength of extruded Ti_2_AlC-AE44 reached 316 MPa. Figure 3b–f presents the different tensile fracture surfaces of specimens. As some Ti_2_AlC particles agglomerated and intermetallics (such as Al_11_Re_3_) spheroidized, the plastic deformation region of as-cast AE44 alloy was strongly reduced with 15 vol.% introduction of Ti_2_AlC, as found in Figure 3b,c. After hot extrusion, when the tensile axis was parallel to the ED axis, Figure 3e shows that some Ti_2_AlC particles were delaminated with the cleavage surface perpendicular to the observed surface. Conversely, delamination of Ti_2_AlC particles with smooth cleavage surfaces was respectively observed in the as-cast composites and in the textured composites when the tensile strength was perpendicular to the ED axis, as shown in Figure 3d,f. This different phenomenon may be a result of the aspect ratio in Ti_2_AlC particles and the extent of particle loading by the shear mechanism [6,23,24,25]. In these studies, the aspect ratio of Ti_2_AlC particles are between 2 and three in this work. The interfacial shear strength between Ti_2_AlC and Mg was subsequently calculated to be around 350–500 MPa, based on the shear mechanism with the aspect ratio of Ti_2_AlC. As shown in Table 1, this range almost contains the maximum tensile strengths in all cases. Herein, no interfacial debonding phenomenon occurred, as shown in Figure 3g,h.

**Figure 4:**
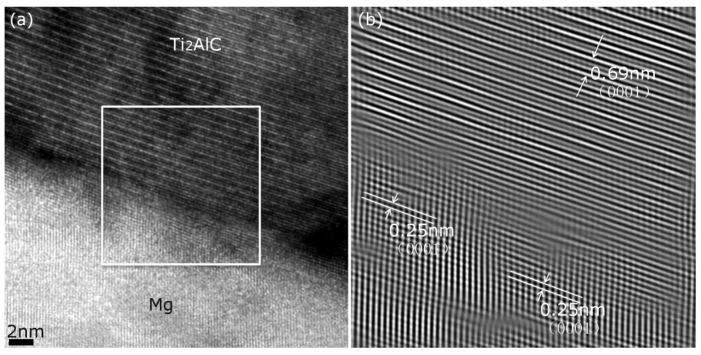
(**a**) High-resolution TEM (HRTEM) micrographs of AE44-Ti_2_AlC interface, (**b**) Fourier transformation of the marked rectangular zone in (**a**).

In addition, the typical HRTEM interface and the Fourier-transformed image shown in Figure 4 indicate that the interfacial lattice transition between Mg and basal plane (0001) of Ti_2_AlC are well-matched, suggesting that both are well-bonded. According to Bramfitt’s equation (1) [38], the potency degree of nucleation catalysts is determined by the average disregistries ($\delta_{{\left(hkl\right)}_{n}}^{{\left(hkl\right)}_{s}}$) along low-index directions within low-index planes between substrate and nucleation solid.

(1) $$\delta_{{\left(hkl\right)}_{n}}^{{\left(hkl\right)}_{s}}=\overset{3}{\underset{i}{\sum }}\left[\left(\left|d_{{\left[uvw\right]}_{s}^{i}}\cos\theta -d_{{\left[uvw\right]}_{n}^{i}}\right|/d_{{\left[uvw\right]}_{n}^{i}}\right)/3\right]\times 100\%$$

where (hkl)_s_ represents a low-index plane of substrate, [uvw]_s_ represents the corresponding low-index direction in (hkl)_s_, d[uvw]_s_ represents the corresponding interatomic spacing along, [uvw]_s_, (hkl)_n_ represent a low-index plane in the nucleation solid, [uvw]_n_ represents the corresponding low-index direction in (hkl)_n_, d[uvw]_n_ represents the corresponding interatomic spacing along [uvw]_n_ and θ represents the angle between [uvw]_s_ and [uvw]_n_. Ti_2_AlC is a hexagonal structure with a and c lattice parameters in 0.30623 and 1.3675 nm. Mg is hexagonal with a and c lattice parameters in 0.32093 and 0.52103 nm.

**Figure 5:**
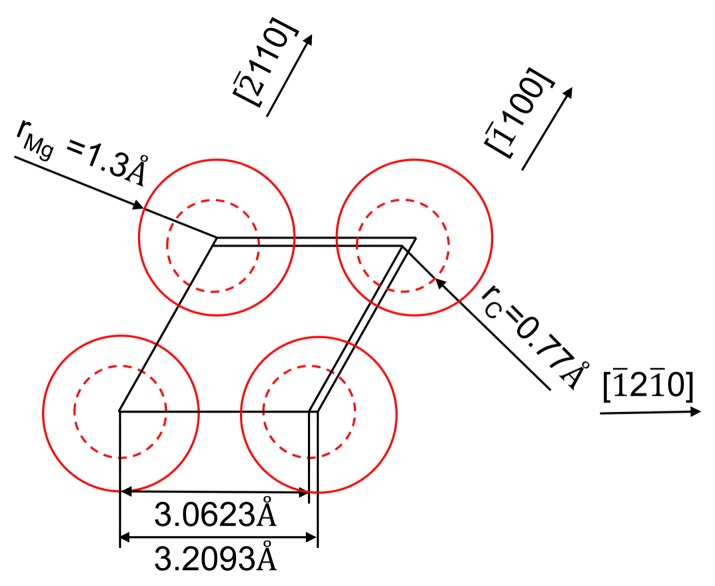
Crystallographic combination of Ti_2_AlC and Mg in (0001) planes (r_Ti_ = 1.36 Å, r_Al_ = 1.18 Å, r_C_ = 0.77 Å, r_Mg_ = 1.3 Å).

Regarding the crystallographic relationships between Mg and Ti_2_AlC, as depicted in Figure 5, the (0001) plane of Mg was overlapped by the (0001) plane of the Ti_2_AlC. By using the lattice parameters and Equation (1), the disregistry between Mg and Ti_2_AlC was calculated as 4.58%, which falls within the “very effective” range for heterogeneous nucleation. If the planar disregistry is less than 12%, the electronic bonding contribution to the energy of the interface is favorable. Therefore, according to this calculation, the crystallographic combination of Ti_2_AlC and Mg in (0001) planes is well-matched, and Ti_2_AlC is believed to be a very potent nucleating substrate for Mg [38,39]. These results further support the experimental observation shown in Figure 5, as well as suggest a strong interface bonding between these two phases. Ti–Al bonds are much weaker than Ti–C bonds in inherent nanolayered structures of Ti_2_AlC [23,26,27]. Consequently, Ti_2_AlC delamination occurred in extruded composites, while no interface debonding occurred.

**Figure 6:**
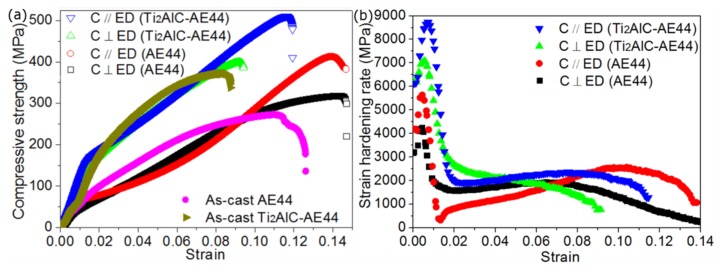
(**a**) Different room-temperature compressive stress–strain curves of as-cast and extruded specimens, (**b**) dσ/dε-ε slope relations derived from stress-strain curves of extruded specimens.

Figure 6a describes the compressive stress–strain curves of as-cast and extruded specimens. It is clear that the introduction of Ti_2_AlC can efficiently enhance the compressive resistance of AE44 alloys. In addition, as found in previous studies [40,41], the anisotropic stress–strain behaviors occurred depending on the compressive axis, especially for extruded Ti_2_AlC-AE44 composites. In order to further reveal the influence of hot extrusion on the performance of AE44 and Ti_2_AlC-AE44 composites, Figure 6b shows the dσ/dε−ε slope relations derived from stress-strain curves. The Ti_2_AlC-AE44 specimen has a clearly higher strain hardening rate than the pure AE44 specimen with respect to relevant directions. After the peak hardening rate, variations of dσ/dε−ε slope for extruded AE44 alloys are different from those of extruded Ti_2_AlC-AE44 composites. In comparison with the curve of C ⊥ ED Ti_2_AlC-AE44, the dσ/dε−ε slope for C // ED Ti_2_AlC-AE44 is characterized by a steady plateau after a peak fast-hardening rate. 

**Figure 7:**
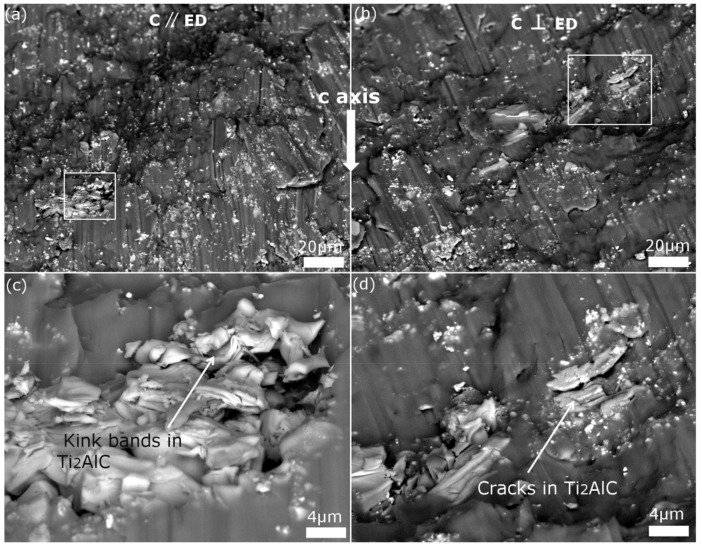
(**a**) and (**b**) Fracture compressive surfaces of the C // ED and C ⊥ ED specimens, (**c**) and (**d**) enlarged area marked by rectangular in (**a**) and (**b**).

As the distributed micro-Ti_2_AlC particles have a strong “pinning effect” in the AE44 matrix, Figure 7a–d shows the severe torn zone appearing above the Ti_2_AlC particles in the glide direction. At the same time, submicro Ti_2_AlC particles and Al_11_Re_3_ were respectively found in shear bands in extruded specimens. Clearly, the magnesium matrix was further strengthened and resulted in a higher strain hardening rate. Furthermore, the differences between Figure 7c,d explain different hardening rates between C // ED and C ⊥ ED for extruded Ti_2_AlC-AE44 composites after the peak of fast hardening rate. The typical kink bands and delamination of Ti_2_AlC MAX phases appeared in C // ED specimens. However, only cracks occurred in some Ti_2_AlC particles for C ⊥ ED specimens.

## 4. Summary

AE44 alloy and nanolaminated-Ti_2_AlC particle-reinforced AE44 magnesium composite were successfully fabricated by stir casting techniques, then textured by hot-extrusion methods. SEM observation revealed that lamellar Al_11_RE_3_ precipitates spheroidized with the introduction of Ti_2_AlC into the AE44 matrix. HRTEM reveals a well-matched interfacial lattice transition between Mg and the basal plane (0001) of Ti_2_AlC. Planar disregistry calculations indicate that the crystallographic combination of Ti_2_AlC and Mg in (0001) planes is well-matched. These results demonstrate that Ti_2_AlC is an effective potent nucleating substrate for Mg, which facilitates the formation of strong interfacial bonds. Herein, no interfacial decohesion occurred in a tensile test. Tensile results indicate the yield strength of the AE44 alloy was enhanced from 88 to 316 MPa with the introduction of 15 vol.% Ti_2_AlC and hot-extrusion treatment. The ultimate compressive strength (UCS) of composites were determined to be 516 (// ED axis) and 394 MPa (⊥ ED), which is higher than the corresponding values (414 and 313 MPa) found in extruded AE44 alloy. This suggests that introduction of Ti_2_AlC and hot extrusion could strongly ameliorate the stiffness of the magnesium matrix. XRD results indicate that extrusion may reorient Ti_2_AlC particles, though it introduces a texture effect on the magnesium matrix. Subsequently, tensile fractures reveal different cleavage orientations of Ti_2_AlC delamination in as-cast and extruded composites. Furthermore, compressive tests reveal the typical kink bands and delamination of Ti_2_AlC MAX phases in C // ED specimens. In contrast, only cracks occurred in some Ti_2_AlC particles for C ⊥ ED specimens. This indicates that the hot extrusion process could regulate the orientation of Ti_2_AlC in AE44 matrix, which could enhance the lubrication capacity of the Ti_2_AlC-AE44-based cylinder wall.

## Figures and Tables

**Table 1:** Summary of mechanical properties of as-cast and hot extruded AE44 alloy and 15 vol.%Ti_2_AlC-AE44 composites. Each category of specimen was repeated 6 times.

Materials	Density (g/cm^3^)	TYS (MPa)	UTS (MPa)	Elongation (%)	UCS (MPa)
As-cast AE44	1.81	88 ± 20	149 ± 20	4.9 ± 0.8	264 ± 5
Extruded AE44	1.83	250 ± 10	397 ± 10	12.2 ± 1.0	414 ± 5 (c // ED axis)
					313 ± 5 (c ⊥ ED axis)
As-cast Ti_2_AlC-AE44	2.14	179 ± 20	200 ± 20	2.6 ± 1.2	371 ± 5
Extruded Ti_2_AlC-AE44	2.16	316 ± 10	416 ± 10	4.4 ± 1.1	516 ± 5 (c // ED axis)
					394 ± 5 (c ⊥ ED axis)
